# Risk Prediction Models for Management of Patients following Acute Aortic Dissection

**DOI:** 10.1055/s-0042-1756671

**Published:** 2022-12-15

**Authors:** Wahaj Munir, Mohamad Bashir, Mohammed Idhrees, Wael I. Awad

**Affiliations:** 1 William Harvey Research Institute, Barts and The London School of Medicine and Dentistry, Queen Mary University of London, United Kingdom; 2 Institute of Cardiac and Aortic Disorders, SRM Institutes for Medical Science (SIMS Hospitals), Chennai, Tamil Nadu, India; 3 Department of Cardiothoracic Surgery, Barts Heart Centre, St. Bartholomew's Hospital, London, United Kingdom

**Keywords:** aorta, aneurysm, dissection, risk prediction, genetics

## Abstract

Risk prediction of adverse outcomes post aortic dissection is dependet not only on the postdissection-associated clinical factors but on the very foundation of the risk factors that lead up to the dissection itself. There are various such risk factors existing prior to the dissection which impact the postdissection outcomes. In this paper, we review the literature to critically analyze various risk models, burdened by their significant limitations, that attempt to stratify risk prediction based on postdissection patient characteristics. We further review several studies across the literature that investigate the diverse set of predissection risk factors impacting postdissection outcomes. We have discussed and appraised numerous studies which attempt to develop a tool to stratify risk prediction by incorporating the impacts of different factors: malperfusion, blood biochemistry, and perioperative outcomes. The well-validated Penn classification has clearly demonstrated in the literature the significant impact that malperfusion has on adverse outcomes postdissection. Other risk models, already severely hindered by their limitations, lack such validation. We further discuss additional alluded risk factors, including the impact of predissection aortic size, the syndromic and nonsyndromic natures of dissection, and the effects of family history and genetics, which collectively contribute to the risk of adverse outcomes postdissection and prognosis. To achieve the goal of a true risk model, there remains the vital need for appreciation and appropriate consideration for all such aforementioned factors, from before and after the dissection, as discussed in this paper. By being able to incorporate the value of true risk prediction for a patient into the decision-making framework, it will allow a new page of precision medical decision-making to be written.

## Introduction

The journey to create a model of stratification for risk prediction of adverse outcomes after the incidence of acute aortic dissection (AD) encompasses an array of factors. 1 2 3 4 5 The goal of such risk models lies in the ability to be incorporated into the decision-making framework for treatment, as well as future care to be provided for the patients and their families.

Numerous studies 1 2 3 4 5 have attempted to create a model to aid in the risk prediction post-AD, in hopes of guiding the surgeon and team in management of their patients. Quantitative and qualitative models have been devised, considering different factors, including varying degrees of malperfusion, blood biochemistry, and perioperative stability, alongside other factors. 1 2 3 4 5 Across these models, the immediate aim is to stratify the risk of operative and 30-day mortality. However, the Penn classification also stratifies the risk of later mortalities up to 5 years. 6 Many of these studies are significantly hindered by intrinsic retrospective limitations and the majority of predictive paradigms still require further validation. 1 2 3 4

When we start to think about risk prediction following AD, how can we ignore the risks that build up prior to the dissection itself? We must consider both syndromic and nonsyndromic cases of AD, as well as familial versus sporadic within the latter data. 7 Risk prediction after the dissection has occurred builds on the very foundation of risk of the initial dissection itself. The family history and known diseases linked to syndromic dissection exert significant influences on the ability to make a prompt diagnosis of AD. Delays in diagnosis and treatment influence the risk of adverse effects postdissection.

This paper reviews the literature to not only critically analyze some of the current models looking to stratify risk prediction postdissection, with their burdening limitations, but also to bring into consideration genetic factors, the natural history of dissection, and the role of prior screening. We explore the potential for these two different, yet clearly related, aspects of risk prediction in AD.

## The Need for Risk Models

A plethora of nonspecific symptoms, an insidious onset, with a lethality rate of 1 to 2% per hour, and a vast range of potential symptoms 8 are just a few of the hurdles challenging an efficient diagnosis of AD. 9 The concomitant events of carotid and cerebral malperfusion worsen the prognosis not only due to poorer clinical conditions of the patients, but also because of the heightened risk of misdiagnosis. 8 10 Any tool to support the efficacy of the decision-making and rapid risk stratification for intervention is invaluable. Stratifying the risk of adverse outcomes postdissection will guide the surgeon toward the optimum therapeutic choice.

## Current Risk Models: Breaking the Code

### Malperfusion

**Fig. 1:**
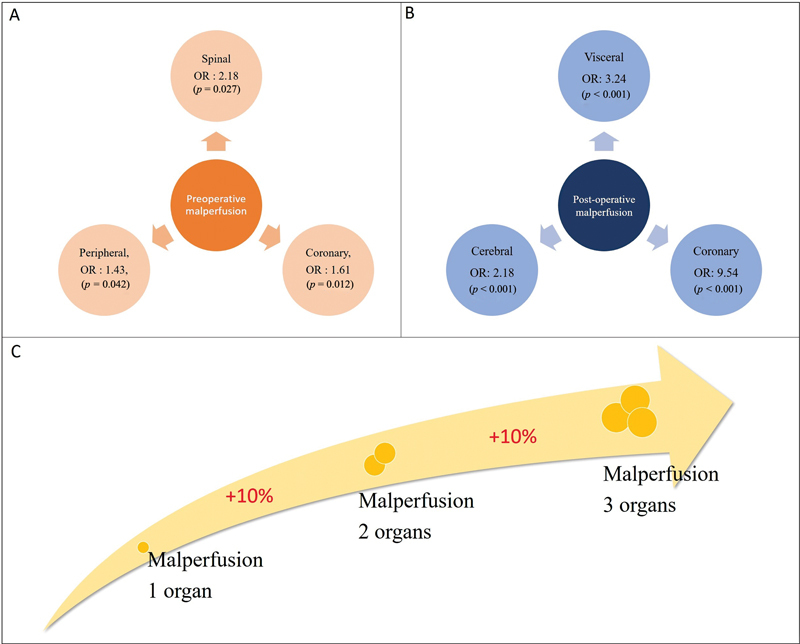
Impact of different types of malperfusion as independent risk factors for early mortality. 7

The impact of malperfusion, with its severe adverse implications, has been well-appreciated, notably illustrated by the German Registry for Acute Aortic Dissection Type A (GERAADA) analysis by Czerny et al 7 ( Fig. 1 ) and the Penn classification. 5

As per the model developed by Augoustide and colleagues, the Penn classification, patients were stratified as 4 levels of ischemia. This classification was defined by authors as follows: class Aa, presence of no ischemia; class Ab, localized ischemia; class Ac, generalized ischemia; and class Abc, localized and general ischemia. 5 The prospective single-centered study analyzing 221 patients, demonstrated an 8.3-fold increase in mortality ( *p* = 0.0001) between patients with no ischemia and patients with any ischemia at all.

The validity of the Penn classification has been substantiated in various retrospective studies 6 11 12 highlighting the significant impact malperfusion has on adverse postdissection outcomes. Table 1 illustrates the impact of malperfusion as an independent risk factor on short- and long-term mortalities as reported across various studies.

**Table 1:** Mortality outcome across the degrees of malperfusion according to Penn's classification 5 6 11 12

		Mortality	Penn's classification				*p* -value
			Aa	Ab	Ac	Abc	
			No ischemia	Localized ischemia	Generalized ischemia	Localized and generalized ischemia	
Original derivation study	Augoustides et al 5 (2005)	All cause	3.1%	25.6%	17.6%	40%	**0.0001** a
		Intraoperative	N/A	N/A	N/A	N/A	N/A
		In-hospital	N/A	N/A	N/A	N/A	N/A
		5 years	N/A	N/A	N/A	N/A	N/A
Validation studies	Kimura et al 6 (2014)	All cause	N/A	N/A	N/A	N/A	N/A
		Intraoperative	3.6%	5.9%	14%	15%	**0.007**
		In-hospital	14%	24%	24%	44%	**0.0007**
		5 years	N/A	N/A	N/A	N/A	N/A
	Olsson et al 11 (2011)	All cause	N/A	N/A	N/A	N/A	N/A
		Intraoperative	N/A	N/A	N/A	N/A	N/A
		In-hospital	3%	6%	17%	22%	< **0.01**
		5 years	15%	26%	22%	33%	Ab: *p* = 0.027
							Abc: *p* < **0.001**
	Pisano et al 12 (2016)	All cause	N/A	N/A	N/A	N/A	N/A
		Intraoperative	N/A	N/A	N/A	N/A	N/A
		In-hospital	10.7%	Non-class Aa: 36.7% b			**0.02**
		5 years	N/A	N/A	N/A	N/A	N/A

Note: N/A, not available.

a *p* = 0.0001 for the comparison of no ischemia versus any ischemia.

b The reported in-hospital mortality by Pisano et al 12 is for collective Penn's class non-Aa versus Aa.

Kimura et al 6 have investigated early and late outcomes following intervention for acute TYPE A aortic dissection (ATAAD), using the Penn risk modeling tool. The authors showed significant ( *p* < 0.01) differences in their results for in-hospital mortality across the different Penn classes: 3 versus 22% for class Aa and Abc, respectively. Further analysis of longer-term outcomes reported significantly lower survival at 5 years for the Penn class Ac and Abc, compared with class A. 6

Upon analysis of a larger group of 360 patients by Olsson et al, 11 the Penn class Abc was validated as a significant independent predictor and risk factor, for intraoperative ( *p* = 0.03) and in-hospital mortality ( *p* = 0.02), respectively. Furthermore, Penn's class non-A ( *p* = 0.014) was in itself an independent risk factor for in-hospital mortality, further highlighting the grave impact of malperfusion. 11

While the Penn classification is well validated, there are limitations to bear in mind. 5 First, building on current validation studies in the literature, there remains a need for prospective validation of the Penn findings in large populations. 5 It is important to consider the hindering limitations of retrospective studies, arising from the lack of recognition of other potential risk factors and their impact on postoperative complications. 11 Furthermore, there is ambiguity in how ischemia was described, a limitation building from the initial Penn's study itself. Also, there is a need for consideration of the vast diversity of clinical manifestations, of ATAAD. 6 Nonetheless some validating studies do provide support for the Penn findings. The distribution of patients amongst classes was similar in validating studies. The significant impact that malperfusion has on outcomes ( Fig. 1 , Table 1 ) is confirmed. 6 11 However, there is still value to be added from consideration of other clinical factors, as our review shall discuss.

### Bringing in the Biochemistry

Ghoreishi et al 1 sought to develop a risk model, building on the premise of the impact of malperfusion, to improve their predicting power for mortality following repair of ATAAD. In their retrospective review at a single institution over a 13-year period, results from 269 patients were included. 1 Following multivariate analysis, they found that creatinine ( *p* = 0.0008), lactic acid ( *p* = 0.01), and liver malperfusion ( *p* = 0.02) were significant risk factors for operative mortality. This risk model attained a c-statistic of 0.75.

Although the authors describe the value of their model, the limitations of their study are acknowledged. The single-centered retrospective nature is one limitation, alongside the need for analysis of long-term results as well as prospective external validation on a larger scale still being required. 1

### International Registry for Acute Aortic Dissection Analysis and Perioperative Factors

With the goal of providing at bedside a tool that can aid the decision-making framework for a surgeon considering an intervention, Rampoldi et al 2 created a risk prediction model for patients undergoing ATAAD repair using their retrospective analysis of 682 patients from the International Registry for Acute Aortic Dissection (IRAD) from 1996 to 2003. Their model encompassed not only preoperative variables but also intraoperative variables as well.

**Fig. 2:**
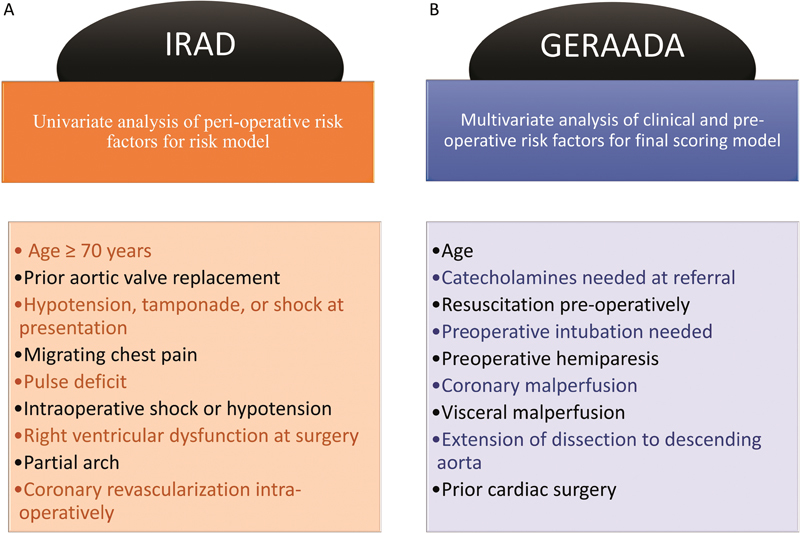
**(A)** Clinical perioperative risk factors used in a risk model devised by Rampoldi et al on their analysis of patients from IRAD. 2 **(B)** clinical preoperative parameters used in the risk model devised by Czerny et al on their analysis of patients from GERAADA. 17 GERAADA, the German Registry for Acute Aortic Dissection Type A; IRAD, International Registry for Acute Aortic Dissection.

Univariate analysis was performed to identify statistically significant clinical characteristics which had an independent impact on operative mortality ( Fig. 2 ).

The authors reported similar significant risk factors of operative mortality as those of prior studies. 2 13 14 The authors point out that their findings should be generalizable, given data collection from various institutions in six countries, with a vast range of clinical presentations taken into consideration. However, their model considers only early mortality. There needs to be further validation of these models, especially for longer-term results. 2

Yu et al 15 analyzed the predictive value of the aforementioned model 2 in a retrospective analysis of a small group of 79 patients. They found their model inadequate in accuracy for risk prediction of surgical outcomes.

### A Scorecard Model

The attempt to create a preoperative “scorecard” for risk prediction of operative mortality was pursued by Leontyev et al. 4 The independent risk factors included age, critical preoperative state, malperfusion syndrome, and coronary artery disease. The authors assigned integer scores for each risk factor depending on the extent of impact; visceral malperfusion would score 3, whereas coronary malperfusion would score 1. 4

With a sample size of 534 consecutive patients, over two institutes with similar patient population characteristics, this model was based on a larger cohort compared with past models. 2 16 Selection bias will always hinder retrospective studies and this is no different for this study, further hampered by a lack of consideration of confounding variables over the study period of almost two decades.

### The German Registry for Acute Aortic Dissection Type A Analysis, Another Scoring Model

A recent multicenter experience of 2,537 patients analyzed from the GERAADA, attempted risk prediction of 30-day mortality. Czerny et al 17 sought to devise a “scoring system” for risk prediction of this early outcome. The final model devised incorporated a variety of clinical and preoperative parameters following multivariate analysis ( Fig. 2 ). This study also overlooked the natural history, genetics, and foundational risks from before the dissection itself. The authors aimed for a “simple, effective tool” 17 for the prediction of only early mortality. The large sample size of patients analyzed from over 50 centers confers power to this study. However, the prediction was found only moderately accurate, with the area under the curve (AUC) of 0.725, lower than The European System for Cardiac Operative Risk Evaluation. A key limitation of GERAADA is the lack of detailed reporting regarding malperfusion and its significant impact on prognosis. Furthermore, there was a lack of consideration for the morphological nature of the tear-tailored surgical approach, which may or may not have been taken in individual cases. 17

**Fig. 3:**
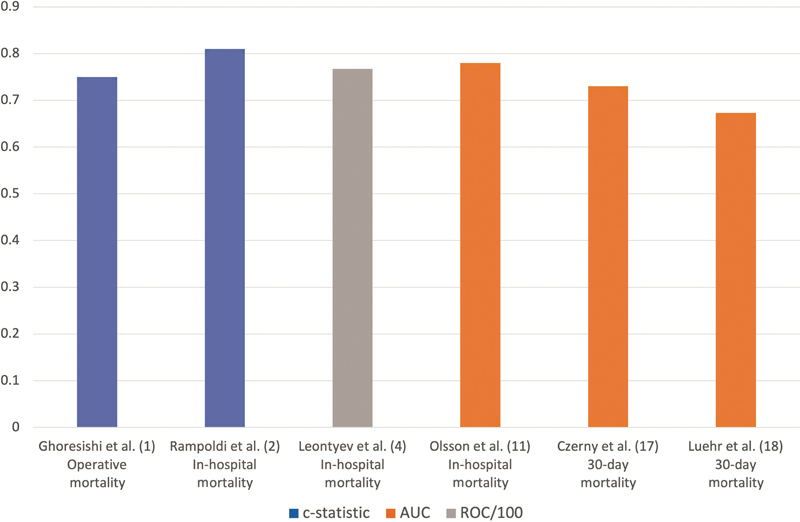
A graph illustrating the AUC and c-statistic scores attained by various risk prediction models in the literature. 1 2 4 11 17 AUC, area under the curve; ROC, receiver operating characteristics.

The GERAADA score devised by Czerny et al 17 has recently been evaluated in a 10-year retrospective study of 371 patients operated on for ATAAD. Luehr et al 18 aimed to investigate if the prediction attained using GERAADA score corresponded with that of the authors' institution results. The authors reported their actual 30-day mortality to be 15.7%, which had been comparable, with no significant difference ( *p* = 0.776), to the predicted mortality of 15.1%. Several patient subgroups had mortality rates with a greater raw difference (higher and lower levels) than those predicted by the GERAADA score; however, these had not reached statistical significance. Luehr et al reported that following multivariable analysis it had been age, resuscitation prior to surgery, and other/unknown malperfusion that were significant independent risk factors for 30-day mortality. The authors use of this risk prediction model attained an AUC score of 0.673. We have illustrated in Fig. 3 , the varying AUC and c-statistic values reported from the respective risk models in the literature. There remains a need for further evaluation of this risk stratification tool in larger scale prospective studies. The authors expressed concerns of over- or underprediction of mortality using the GERAADA score, especially for smaller subgroups of less than 100 patients where there had been notable raw differences in actual vs predicted rates, albeit not statistically significant. 18

## The Problem at Hand

We need a predictive tool that functions with ease, accuracy, and precision, despite the inherent limitations that often hinder such studies. Currently, due to limitations, we have only half a canvas.

## Exploring the Natural History: The Missed Elements

### The Upcoming Dissection, Does Size Matter?

A study reporting on the Yale database 19 analyzing results of 304 patients from 1985 to 2000 concluded that greater risks of rupture and dissection are linked to the greater initial size of the aorta ( *p* = 0.006). An aorta being >6 cm is reported as three times worse in comparison to sizes of 4 to 4.9 cm. Davies et al 19 further reported that long-term survival was lower for patients with greater-sized aneurysms ( *p* = 0.0039).

Although the relatively large sample size along with long follow-up periods gives strength to this study, which provided great insight into the natural history and risk factors for rupture or dissection, patients followed in this single-centered retrospective study were operated on electively based on criteria of aortic size, hence not permitting prediction of postdissection outcomes. The reported yearly rates may in fact be a representation of the lower limits of the more accurate rates, as discussed by the authors. 19

### The Genetic Factors to Consider

Greater than 90% of thoracic aortic aneurysms are asymptomatic prior to dissection, with less than half being diagnosed appropriately in emergency departments prior to patient death. 20 Furthermore, considering that approximately a fifth of dissection patients have a corresponding family history, the importance of investigating genetic links is amplified. 21 22

The build-up to both syndromic and nonsyndromic onsets can potentially boil down to a single mutated gene as the causative factor. 21 The syndromic side is better defined, resultant of our stronger understanding of the interactions between aortic pathologies and connective tissue disorders such as the Loeys–Dietz, Marfan, and Ehlers–Danlos syndromes. 21 23 Pathogenesis involves “dysfunction of the extracellular matrix, medial smooth muscle cells, or TGF-β signaling.” 21 Recognition of these syndromic cases may require more aggressive and extensive replacement approaches. 24 25

It was initially thought that the rest of the patients without a family history, these so-termed “sporadic” cases, were purely degenerative conditions; however, research has suggested that there may well be an underlying genetic mechanism. 26 Even in patients with sporadic AD, pathogenic genetic variants found are in fact common with those of syndromic features. One such example is a variant of the *FBN1* gene, with its causative link in Marfan syndrome, which can also be a risk factor for sporadic cases. 21 Guo et al 26 found that 9.3% of patients with sporadic thoracic AD in fact had pathogenic variants in heritable genes. Genetic screening may well benefit not just the patient but also their families, as further discussed by Ostberg et al. 21

**Fig. 4:**
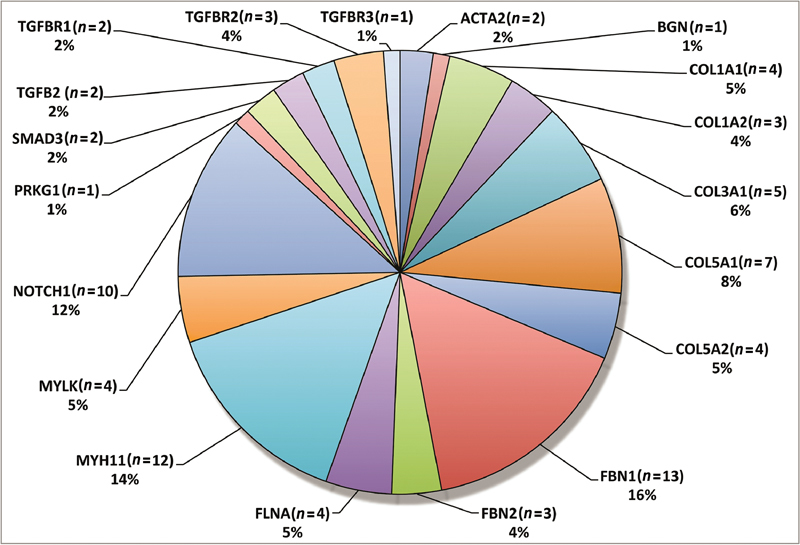
Frequency distribution of genetic defects in thoracic aortic aneurysm and dissection—related genes as per the routine genetic testing program at the Yale Aortic Institute. Reproduced with permission from Vinholo et al. 29

As recently as 2017, there were only 29 identified genes associated with the development of AD. 27 In the latest updates from 2019, there are now 37 identified genes for this association. 28 These genes still only explain approximately 30% of familial nonsyndromic TAAD. 26 Fig. 4 illustrates the distribution of the aforementioned genes. 29

Bearing these thoughts in mind and the progressive speed with which the “genetic dictionary” will continue to evolve, alongside our evolving understanding of the genetic foundation and natural history of AD will allow a new page to be written in the field of personalized precision medicine. 28

### Family History and Prognosis

Genetic factors have an impact on the heritability and incidence of AD. 26 27 28 However, a key aspect is the impact that the presence of AD in the family history may have on prognostic outcomes.

Chen et al 30 hypothesized an association between the presence of family history and the prognosis of AD. In their nationwide study, a total of 93 AD patients with a family history were matched with 894 control AD patients without any family history. The propensity-score matching process encompassed: “age at diagnosis of AD, sex, comorbidities, and medication.” 30 The authors reported no significant difference between the groups for rates of in-hospital mortality. There may have been higher incidences of root replacement procedures for ATAAD patients that had a family history. It was found that for patients in the family history group, there was a significantly higher risk of the patients having to receive later aortic surgical intervention. 30

Despite the strengths of this prospective (from the National Health Insurance Research Database), we cannot ignore some key limitations. The potential misclassification of diagnosis, lack of anatomic imaging in the database, and difficulty in generalizing these conclusions based on results from the Taiwanese population are notable weaknesses. Furthermore, there was a stark difference in the number of patients in the compared groups. 30

### The Unfortunate Impact of Delays

A plethora of nonspecific symptoms, an insidious onset, and a vast range of symptoms often lead to delay in diagnosis of AD, 8 especially distressing given the mortality rate of 1 to 2% per hour. 9 The concomitant events of carotid and cerebral malperfusion carry the burden of a worse prognosis, not only due to poorer clinical conditions of the patients but due to the heightened risk of misdiagnosis. 8 10

Harris et al 31 reported from their analysis of data from IRAD that those patients who had undergone previous cardiac surgery, were transferred from nontertiary centers of non-White race, or female had significantly greater time taken from the presentation to diagnosis.

Although extending transport times, there remain significant advantages of transferring patients to high-volume centers. Goldstone et al reported that there was in fact a 7.2% reduction in risk of operative mortality for patients transferred to high-volume centers. 32 Our recent analysis of 249 patients, from the National Institute for Cardiovascular Outcomes Research database, further concluded that greater levels of in-hospital mortality are related to lower volume surgeons. 33

## Conclusion… Paving the Future

We have discussed the need for consideration and appreciation of the significance carried by factors both prior to and following AD and how a true risk prediction model of adverse outcomes postdissection can only be developed with due respect for all these factors surrounding AD.

A greater aortic size not only predicts the risk of dissection occurring but carries with it a greater risk of worse long-term outcomes, amplifying its importance in the decision-making framework. 19 There is recognition of the impact that connective tissue disorders have on the management for AD; however, even sporadic cases of dissection can have pathogenic variants of heritable genes such as *FBN1* . 21 Recognition of the many genes associated with AD eloquently highlights the importance of considering the genetic foundation that is painting the picture and the need for its incorporation in the decision-making framework. 28

The Penn classification has been recognized as a valuable tool for risk stratification for AD. Despite some significant limitations of the validating studies, there is strong encouragement and verification of this classification as a risk model. 5 6 11 12 There are various other risk models, yet to be validated, proposed in the literature, showing that fellow perioperative factors, blood biochemistry results, and comorbidities all have a role in risk prediction ( Table 2 ).

**Table 2:** Risk prediction of adverse outcomes after acute aortic dissection: key messages, points, and conclusions

Key summary points
• Various studies have devised their own models for the stratification of risk prediction in acute aortic dissection. These have significant inherent limitations, and majority of the models lack large-scale prospective external validation
• The well-validated Penn classification demonstrates the incorporation of varying levels of malperfusion as a significant factor in a risk prediction model
• Many of these risk prediction models following dissection overlook the influence of predissection risk and patient characteristics
• Malperfusion, blood biochemistry, and clinical perioperative factors are significant components for a risk prediction model; however, they make up one end of the spectrum.
• Impact of genetics in syndromic and nonsyndromic cases are fundamental elements. Further appreciation for aortic size and the impact of delays in transport and diagnosis are significant covariates for postdissection outcomes
• We must aim toward personalized precision medicine, which is only achieved by incorporating all risk prediction components into the decision-making framework

We have discussed in our recent paper how risk profiling, optimum assessment, and increased awareness for AD will facilitate diagnosis and efficient transition of patients from ER to OR. This will further reduce the risk of postdissection mortality. 34

This journey to develop a stratification tool for the risk of adverse outcomes once the dissection has occurred builds atop the very foundation of risk prior to the dissection itself. We must appreciate the classification of the dissection regarding syndromic and nonsyndromic, with recognition of family history, understanding of the natural history with the immense genetic impact, alongside the vital role those clinical characteristics play. Only following the incorporation of all these various aspects can we begin to put the puzzle together and have a true risk prediction model to consider for our patients.
